# A Single Residue in Ebola Virus Receptor NPC1 Influences Cellular Host Range in Reptiles

**DOI:** 10.1128/mSphere.00007-16

**Published:** 2016-03-30

**Authors:** Esther Ndungo, Andrew S. Herbert, Matthijs Raaben, Gregor Obernosterer, Rohan Biswas, Emily Happy Miller, Ariel S. Wirchnianski, Jan E. Carette, Thijn R. Brummelkamp, Sean P. Whelan, John M. Dye, Kartik Chandran

**Affiliations:** a Department of Microbiology and Immunology, Albert Einstein College of Medicine, Bronx, New York, USA; b United States Army Medical Research Institute of Infectious Diseases, Fort Detrick, Maryland, USA; c Department of Microbiology and Immunobiology, Harvard Medical School, Boston, Massachusetts, USA; d Netherlands Cancer Institute, Amsterdam, The Netherlands; e Department of Microbiology and Immunology, Stanford University School of Medicine, Stanford, California, USA; Boston University School of Medicine

**Keywords:** Ebola virus, NPC1, Niemann-Pick C1, endosomal receptor, filovirus, intracellular receptor, reptiles, viral receptor, virus-host interactions

## Abstract

Filoviruses are the causative agents of an increasing number of disease outbreaks in human populations, including the current unprecedented Ebola virus disease (EVD) outbreak in western Africa. One obstacle to controlling these epidemics is our poor understanding of the host range of filoviruses and their natural reservoirs. Here, we investigated the role of the intracellular filovirus receptor, Niemann-Pick C1 (NPC1) as a molecular determinant of Ebola virus (EBOV) host range at the cellular level. Whereas human cells can be infected by EBOV, a cell line derived from a Russell’s viper (*Daboia russellii*) (VH-2) is resistant to infection in an NPC1-dependent manner. We found that VH-2 cells are resistant to EBOV infection because the Russell’s viper NPC1 ortholog bound poorly to the EBOV spike glycoprotein (GP). Analysis of panels of viper-human NPC1 chimeras and point mutants allowed us to identify a single amino acid residue in NPC1, at position 503, that bidirectionally influenced both its binding to EBOV GP and its viral receptor activity in cells. Significantly, this single residue change perturbed neither NPC1’s endosomal localization nor its housekeeping role in cellular cholesterol trafficking. Together with other recent work, these findings identify sequences in NPC1 that are important for viral receptor activity by virtue of their direct interaction with EBOV GP and suggest that they may influence filovirus host range in nature. Broader surveys of *NPC1* orthologs from vertebrates may delineate additional sequence polymorphisms in this gene that control susceptibility to filovirus infection.

**IMPORTANCE** Identifying cellular factors that determine susceptibility to infection can help us understand how Ebola virus is transmitted. We asked if the EBOV receptor Niemann-Pick C1 (NPC1) could explain why reptiles are resistant to EBOV infection. We demonstrate that cells derived from the Russell’s viper are not susceptible to infection because EBOV cannot bind to viper NPC1. This resistance to infection can be mapped to a single amino acid residue in viper NPC1 that renders it unable to bind to EBOV GP. The newly solved structure of EBOV GP bound to NPC1 confirms our findings, revealing that this residue dips into the GP receptor-binding pocket and is therefore critical to the binding interface. Consequently, this otherwise well-conserved residue in vertebrate species influences the ability of reptilian NPC1 proteins to bind to EBOV GP, thereby affecting viral host range in reptilian cells.

## Abstract (precis)

Identifying cellular factors that determine susceptibility to infection can help us understand how Ebola virus is transmitted. We asked if the EBOV receptor Niemann-Pick C1 (NPC1) could explain why reptiles are resistant to EBOV infection. We demonstrate that cells derived from the Russell’s viper are not susceptible to infection because EBOV cannot bind to viper NPC1. This resistance to infection can be mapped to a single amino acid residue in viper NPC1 that renders it unable to bind to EBOV GP. The newly solved structure of EBOV GP bound to NPC1 confirms our findings, revealing that this residue dips into the GP receptor-binding pocket and is therefore critical to the binding interface. Consequently, this otherwise well-conserved residue in vertebrate species influences the ability of reptilian NPC1 proteins to bind to EBOV GP, thereby affecting viral host range in reptilian cells.

## INTRODUCTION

Ebola virus (EBOV) is the causative agent of highly lethal zoonotic infections in humans and nonhuman primates in sub-Saharan Africa (1–3). Despite the emerging roles of EBOV and related members of the family *Filoviridae* (filoviruses) in human disease, our knowledge of the ecological host range of these agents remains limited. Bats are thought to be important reservoirs for filoviruses; however, conclusive evidence in favor of this hypothesis has been obtained only for Marburg virus (MARV) and Ravn virus (RAVV), which were recently found to circulate in Egyptian rousettes (*Rousettus aegyptiacus*) (4–7).

Previous studies demonstrated that, whereas a broad range of mammalian and avian cell lines are susceptible to EBOV and/or MARV, all tested reptilian and amphibian lines are resistant to infection (8–10). These findings suggested the existence of one or more unknown determinants of filovirus host range. Although the determinants of filovirus infection and disease at the organismal level are likely to be complex, it is well established that interactions between viruses and cell-intrinsic host factors, such as entry receptors, can dictate host range. For example, ortholog-specific sequence variations in angiotensin-converting enzyme 2 (ACE2) and transferrin receptor (TfR1) influence the host range of viruses for which they serve as receptors (severe acute respiratory syndrome-related coronaviruses [11, 12] and New World mammarenaviruses, canine parvoviruses, and murine mammary tumor virus [13–18], respectively). Jae and coworkers demonstrated that chicken cells are resistant to infection by an Old World arenavirus, Lassa virus, because of a single amino acid difference in the chicken ortholog of its intracellular receptor, LAMP1 (19).

We and others recently demonstrated that Niemann-Pick C1 (NPC1), a large endo/lysosomal membrane protein involved in cellular cholesterol trafficking, is an essential intracellular receptor for filovirus entry and infection (20–23). We also found that NPC1 could influence the cellular host range of filoviruses—human NPC1 conferred susceptibility to filovirus entry and infection when expressed in the nonpermissive reptilian cell line VH-2, derived from a Russell’s viper (*Daboia russellii*) (22). In that study, however, we did not establish the molecular basis of the NPC1-dependent block to viral entry in VH-2 cells.

Recently, we found that a single amino acid residue (position 502) in the second luminal domain of NPC1, domain C, is under positive selection in bats and controls the susceptibility of bat cells to EBOV infection in a host species-dependent manner (24, 25). Here, we demonstrate that an adjacent residue, 503, highly conserved in domain C of NPC1, influences EBOV host range in reptilian cells by controlling its activity as a filovirus receptor. The recently solved structure of the EBOV entry glycoprotein (GP_1,2_; hereafter referred to as GP) bound to domain C shows that these two residues are in a loop that dips into the exposed receptor-binding site (26). Therefore, our findings identify a hot spot in NPC1 at the EBOV GP-binding interface that influences virus-receptor recognition and host cell susceptibility, suggesting evolutionary scenarios in which antagonism with filoviruses could sculpt host *NPC1* genes selectively, without compromising their ancient, and essential, function in cellular cholesterol homeostasis.

## RESULTS

### The second luminal domain of the Russell’s viper NPC1 ortholog binds poorly to the Ebola virus glycoprotein.

We postulated that EBOV fails to enter and infect Russell’s viper VH-2 cells because the EBOV entry glycoprotein, GP, cannot recognize the viper ortholog of the filovirus intracellular receptor, Niemann-Pick C1 (*Daboia russellii* NPC1 [*Dr*NPC1]). We previously showed that the second luminal domain (C) of human NPC1 (*Homo sapiens* NPC1 [*Hs*NPC1]) directly contacts a cleaved form of EBOV GP (GP_CL_) and that GP_CL_-*Hs*NPC1 domain C binding is essential for filovirus entry (22, 23). Accordingly, we investigated the capacity of *Dr*NPC1 domain C to bind to GP_CL_ and support EBOV entry and infection.

**FIG 1:**
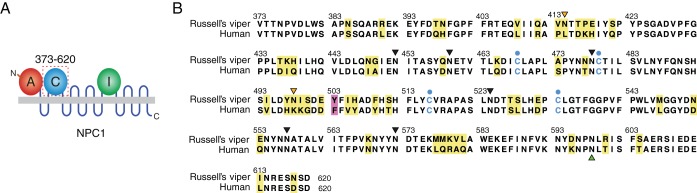
Alignment of human and viper NPC1 domains C. (A) Schematic of full-length NPC1 protein, showing luminal domains A, C, and I. (B) Alignment of NPC1 domain C sequences from human and Russell’s viper. Cysteine residues are in blue. Predicted N-glycosylation sites (sequons) that are conserved in the two proteins are indicated with black arrowheads. Orange arrowheads mark those unique to Russell’s viper NPC1 domain C, and a green arrowhead marks one that is unique to human NPC1 domain C. Nonidentical residues are highlighted in yellow. Position 503 is highlighted in pink.

We first used reverse transcription-PCR (RT-PCR) to isolate and sequence *Dr*NPC1 domain C. Alignment of domain C amino acid sequences from *Hs*NPC1 and *Dr*NPC1 revealed a substantial degree of conservation (80% amino acid identity), with identical arrangements of cysteine residues and similar predicted secondary structures, suggesting a similar overall fold for the two proteins (Fig. 1).

**FIG 2:**
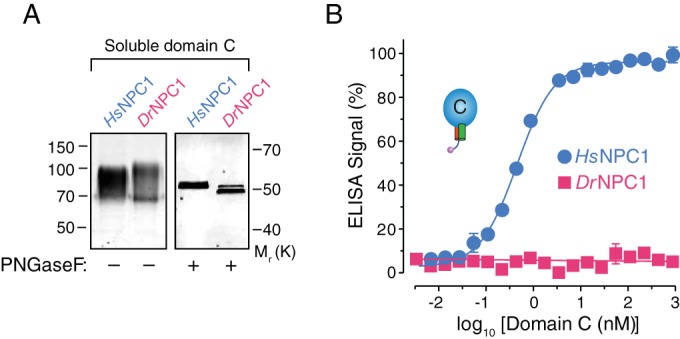
Both *Hs*NPC1 and *Dr*NPC1 domain C proteins are expressed and secreted but bind differentially to EBOV GP_CL_. (A) Soluble forms of the NPC1 domain C proteins from human (*Hs*NPC1) and Russell’s viper (*Dr*NPC1) were expressed in FreeStyle 293-F cells and purified by nickel-affinity chromatography. Equal concentrations were resolved by anti-Flag immunostaining. Left, no treatment. Right, treatment with protein *N*-glycosidase F (PNGase F). Numbers at left are molecular masses in kilodaltons, and numbers at right are relative molecular weights in thousands. (B) The two NPC1 domain C proteins were tested in an ELISA for binding to EBOV GP_CL_. VSV-EBOV GP viruses were cleaved with thermolysin (250 µg/ml) and captured on an ELISA plate using monoclonal antibody KZ52. Serial dilutions of either *Hs*NPC1 or *Dr*NPC1 domain C proteins were added, and binding to GP_CL_ was detected by anti-Flag antibody.

To facilitate *in vitro* GP_CL_-NPC1-binding studies, we engineered a soluble form of *Dr*NPC1 domain C, as previously described for *Hs*NPC1 (22). Transfection of HEK 293T cells with this construct afforded the secretion of an extensively N-glycosylated form of *Dr*NPC1 domain C (Fig. 2A). As shown previously, purified *Hs*NPC1 domain C could bind to recombinant vesicular stomatitis virus Indiana particles bearing cleaved EBOV GP (rVSV-GP_CL_), as measured by enzyme-linked immunosorbent assay (ELISA) (22); in contrast, *Dr*NPC1 domain C exhibited no binding by ELISA, even at the highest concentration tested (Fig. 2B). Therefore, *Dr*NPC1 domain C, in contrast to its human counterpart, recognizes the EBOV glycoprotein poorly or not at all.

### *Dr*NPC1 domain C can substitute for *Hs*NPC1 domain C in mediating endo/lysosomal cholesterol clearance but not EBOV entry and infection.

**FIG 3:**
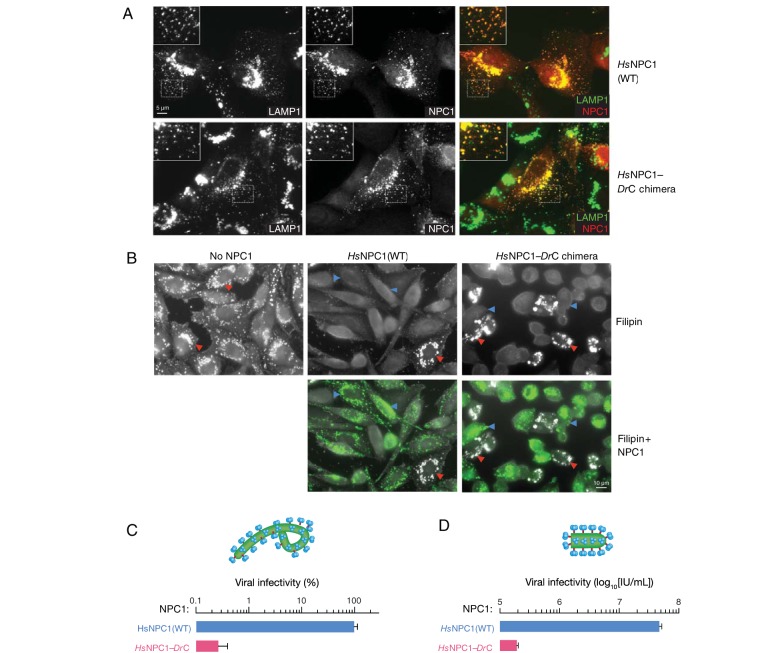
*Hs*NPC1-*Dr*C chimera is functional at cholesterol clearance from lysosomes but does not support EBOV entry and infection. (A) Full-length NPC1 constructs—human WT (*Hs*NPC1) and the human NPC1 chimera with the domain C replaced with viper domain C (*Hs*NPC1-*Dr*C)—immunostained with anti-Flag antibody (red) colocalize with the lysosomal marker LAMP1 (green) when transiently expressed in U2OS NPC1^−/−^ cells (27). (B) CHO-M12 cells stably expressing either *Hs*NPC1 WT or *Hs*NPC1-*Dr*C were stained with filipin to visualize unesterified cholesterol. Top panel, filipin staining. Cholesterol-laden cells are marked with red arrowheads. Blue arrowheads indicate cells that are functional at cholesterol clearance. Bottom panel, cells immunostained with anti-Flag antibody for NPC1 expression (green). (C) Infection of cells from panel B by authentic EBOV (multiplicity of infection of 10), scored 72 h postinfection and normalized to infection on *Hs*NPC1(WT). (D) Infection of cells from panel B by VSV-EBOV GP calculated by manual counting of eGFP-positive cells. IU/ml, infectious units per milliliter. Means ± standard deviations (*n* = 2 to 4) from a representative experiment are shown in each panel.

While the efficient secretion of the soluble, glycosylated *Dr*NPC1 domain C construct suggested that it was not misfolded, it was nevertheless conceivable that subtle structural aberrations rendered this protein biologically inactive. Accordingly, we assessed the capacity of *Dr*NPC1 domain C to support NPC1’s best-established cellular function—clearance of unesterified cholesterol from endo/lysosomal compartments (Fig. 3A and B). This activity requires the full-length NPC1 protein, including all three major luminal domains, A, C, and I. We therefore generated and tested an *Hs*NPC1 chimera in which domain C (residues 373 to 620) was seamlessly replaced with its viper counterpart (*Hs*NPC1-*Dr*C)—we previously found that replacing domain C in NPC1 using restriction site cloning, which introduced two additional amino acid residues at each junction, resulted in proteins that were defective in localization and cholesterol clearance. The wild-type (WT) *Hs*NPC1 and *Hs*NPC1-*Dr*C chimera constructs were then stably expressed in the NPC1-null Chinese hamster ovary (CHO) M12 cell line (24). As expected, immunostaining of WT *Hs*NPC1 transiently expressed in a U2OS NPC1^−/−^ cell line (27) showed colocalization with the endo/lysosomal marker LAMP1 (Fig. 3A) The behavior of *Hs*NPC1-*Dr*C resembled that of WT *Hs*NPC1, indicating that it too localizes to endo/lysosomal compartments (Fig. 3A). These results suggest that *Dr*NPC1 domain C is correctly folded and does not interfere with the correct folding and trafficking of full-length *Hs*NPC1.

We next monitored the cholesterol clearance activity of each protein upon stable expression in NPC1-null M12 cells (Fig. 3B). Filipin, a fluorescent probe for free cholesterol, extensively stained the cholesterol-laden endo/lysosomal compartments of the parental M12 cells, as shown previously (24). Ectopic *Hs*NPC1 expression could clear this accumulated cholesterol, as previously described (20), substantially reducing filipin staining. Remarkably, *Hs*NPC1-*Dr*C could rescue cholesterol clearance as efficiently as WT *Hs*NPC1 (Fig. 3B). These findings affirm that *Dr*NPC1 domain C is biologically active and competent to perform a major housekeeping function of its human counterpart, despite its divergence from the latter at 48 out of 248 amino acid positions (Fig. 1).

Finally, we challenged M12 cell lines expressing WT *Hs*NPC1 or *Hs*NPC1-*Dr*C with authentic EBOV (Fig. 3C). Replacement of human domain C with its Russell’s viper ortholog reduced EBOV infection by almost 3 orders of magnitude. Similar results were obtained in infections with rVSV-EBOV GP (Fig. 3D), confirming that the *Dr*NPC1 domain C-imposed infection block occurs at the viral entry step. Taken together, these observations afford two conclusions. First, the failure of *Dr*NPC1 to support EBOV entry and infection arises at least in part because its domain C cannot bind to EBOV GP_CL_. Second, one or more differences between the domain C sequences of *Hs*NPC1 and *Dr*NPC1 render *Dr*NPC1 bereft of viral receptor activity without perturbing its normal function in cellular cholesterol homeostasis.

### Differences in N-glycosylation do not explain the defect in EBOV GP_CL_-*Dr*NPC1 domain C binding.

**FIG 4:**
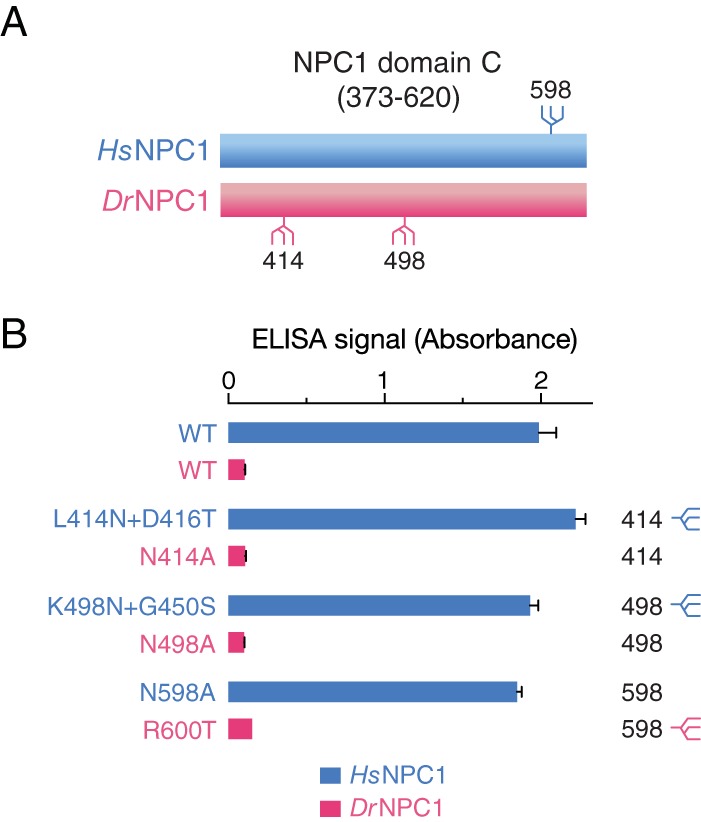
N-glycosylation of NPC1 domain C does not affect EBOV GP_CL_ binding. (A) Location of the three unique sequons in *Hs*NPC1 versus *Dr*NPC1 domain C. (B) Glycosylation mutants were made in both *Hs*NPC1 (losing sequon at position 598 and gaining sequons at position 414 and 498) and *Dr*NPC1 (losing sequons at position 414 and 498 and gaining sequon at position 598). Domain C proteins were expressed in HEK 293T cells and tested for EBOV GP_CL_ binding by ELISA.

To uncover the molecular basis of *Dr*NPC1’s defective viral receptor function, we engineered and tested a panel of mutant, soluble NPC1 domain C constructs in both *Hs*NPC1 and *Dr*NPC1 backgrounds. We first considered the possibility that one or more differences in N-linked glycosylation sites determine the *Hs*NPC1-*Dr*NPC1 difference, because it is either required for GP_CL_-*Hs*NPC1 binding or deleterious for GP_CL_-*Dr*NPC1 binding (Fig. 4). Six sequons are conserved between the two proteins, but *Dr*NPC1 and *Hs*NPC1 domains C contain two and one unique sequons, respectively. Accordingly, we generated soluble domain C proteins containing or lacking each unique sequon and tested these putative gain-of-function and loss-of-function mutants for binding to EBOV GP_CL_. “Humanized” *Dr*NPC1 domain C proteins engineered to lack their unique sequons at position 414 or 498 (*Hs*NPC1 numbering) or to gain the sequon at position 598 remained defective at EBOV GP_CL_ binding in the ELISA. Conversely, *Hs*NPC1 domain C proteins engineered to resemble *Dr*NPC1 at each of these three positions remained fully competent to bind to EBOV GP_CL_. Therefore, differences in N-linked glycosylation between the domains C of *Hs*NPC1 and *Dr*NPC1 do not account for the defective EBOV receptor activity of *Dr*NPC1.

### A single point mutation renders *Dr*NPC1 domain C competent to bind to EBOV GP_CL_.

**FIG 5:**
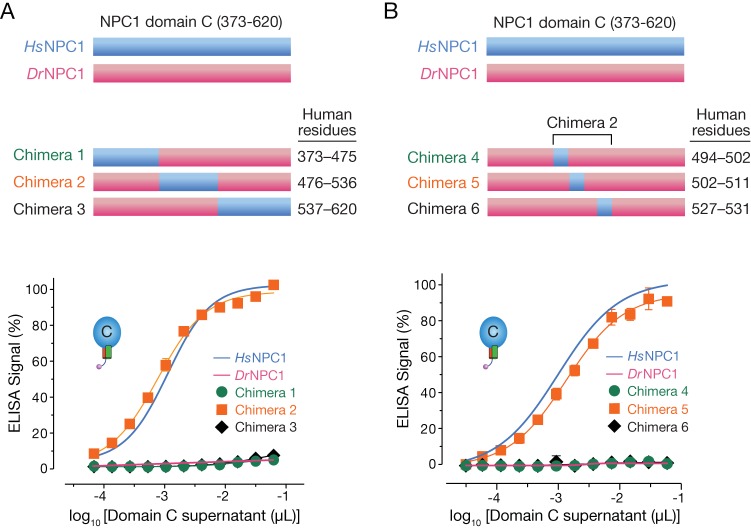
Middle region of *Hs*NPC1 domain C confers binding ability on *Dr*NPC1. (A) Chimeras were engineered by replacing *Dr*NPC1 domain C sequences with human sequence 373 to 475 (chimera 1), 476 to 536 (chimera 2), or 537 to 620 (chimera 3). The chimeras were expressed in HEK 293T cells and tested for EBOV GP_CL_ binding by ELISA. (B) Further dissection of chimera 2 was done by replacing smaller subsets of *Dr*NPC1 with human residues 494 to 502 (chimera 4), 502 to 511 (chimera 5), and 527 to 531 (chimera 6). Chimeric NPC1 domain C proteins were tested as in panel A.

Having ruled out a role for variations in N-glycosylation, we next adopted a systematic approach to identify determinative sequences in NPC1 domain C. We expressed a series of soluble *Hs*NPC1-*Dr*NPC1 domain C chimeras and measured their activity in the GP_CL_-binding ELISA (Fig. 5). However, only chimera 2, *Dr*NPC1 domain C containing *Hs*NPC1 residues 476 to 536, afforded GP_CL_-NPC1 binding (Fig. 5A).

Chimera 2 introduced 14 Russell’s viper→human amino acid changes into *Dr*NPC1. To further dissect their roles, we generated and tested three additional chimeras containing subsets of these amino acid changes (chimeras 4 to 6, Fig. 5B) in the GP_CL_-binding ELISA. The subregion chimera 5 fully reconstituted GP_CL_-*Dr*NPC1 domain C binding, providing evidence that one or more of the 6 *Hs*NPC1 residues in this construct confer gain of function on *Dr*NPC1.

**FIG 6:**
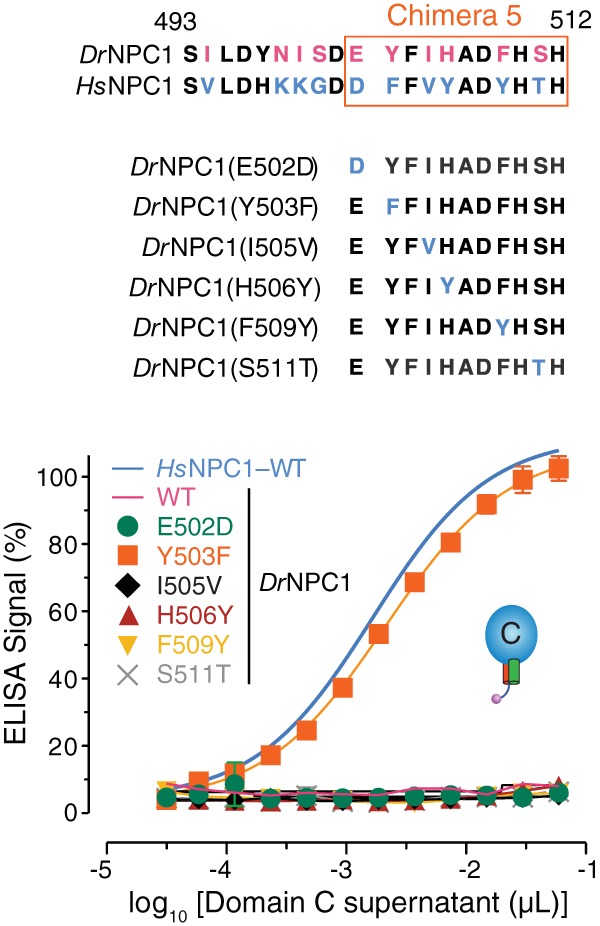
A single amino acid change renders *Dr*NPC1 domain C fully competent to bind EBOV GP_CL_. Chimera 5 contains 6 amino acid differences between *Dr*NPC1 and *Hs*NPC1 domain C. The following point mutations were made in the *Dr*NPC1 domain C by switching the viper amino acid residue at each of these positions to the corresponding human residue: E502D, Y503F, I505V, H506Y, F509Y, and S511T. The point mutants were expressed in HEK 293T cells and tested for EBOV GP_CL_ binding by ELISA.

To assess the individual contributions of the six Russell’s viper→human amino acid changes in chimera 5, we separately introduced these changes into soluble *Dr*NPC1 domain C and tested the capacity of each point mutant to bind to EBOV GP_CL_ (Fig. 6). A single conservative mutation, Y503→F, fully restored GP_CL_-*Dr*NPC1 domain C binding, whereas the other 5 mutations had no discernible effect. Thus, the presence of Y instead of F at position 503 appears to completely explain the failure of *Dr*NPC1 to bind to EBOV GP_CL_.

### F↔Y sequence change at residue 503 controls NPC1’s function as an EBOV entry receptor without affecting its housekeeping function.

**FIG 7:**
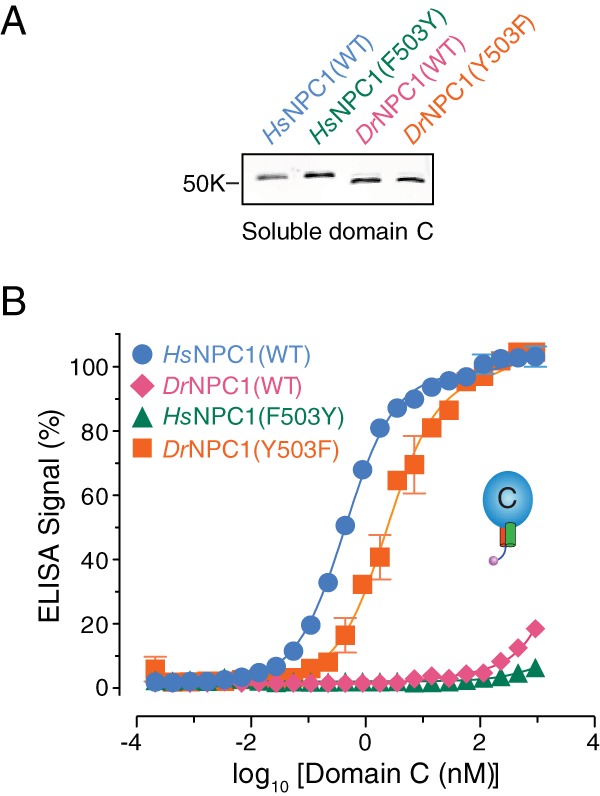
NPC1 residue 503 bidirectionally alters domain C’s capacity to bind EBOV GP_CL_. (A) *Hs*NPC1 and *Dr*NPC1 domain C proteins bearing point mutations at residue 503 (*Hs*NPC1, F503Y; *Dr*NPC1, Y503F) were expressed and purified. (B) Serial dilutions of equivalent amounts of purified NPC1 domain C proteins were tested for EBOV GP_CL_ binding by ELISA.

We postulated that the F↔Y sequence change at residue 503 might influence EBOV GP_CL_-NPC1 binding in a bidirectional manner. Accordingly, we expressed and purified the reciprocal *Dr*NPC1(Y503F) and *Hs*NPC1(F503Y) domain C mutants and tested them in the GP_CL_-binding ELISA (Fig. 7). Purified *Dr*NPC1(Y503F) domain C bound almost as well as its human counterpart to EBOV GP_CL_ (50% effective concentration [EC_50_] for binding, ≈3 nM [Russell’s viper] versus 0.5 nM [human]). Conversely, no detectable GP_CL_ binding was obtained with the *Hs*NPC1(F503Y) domain C protein (EC_50_, >1 µM).

**FIG 8:**
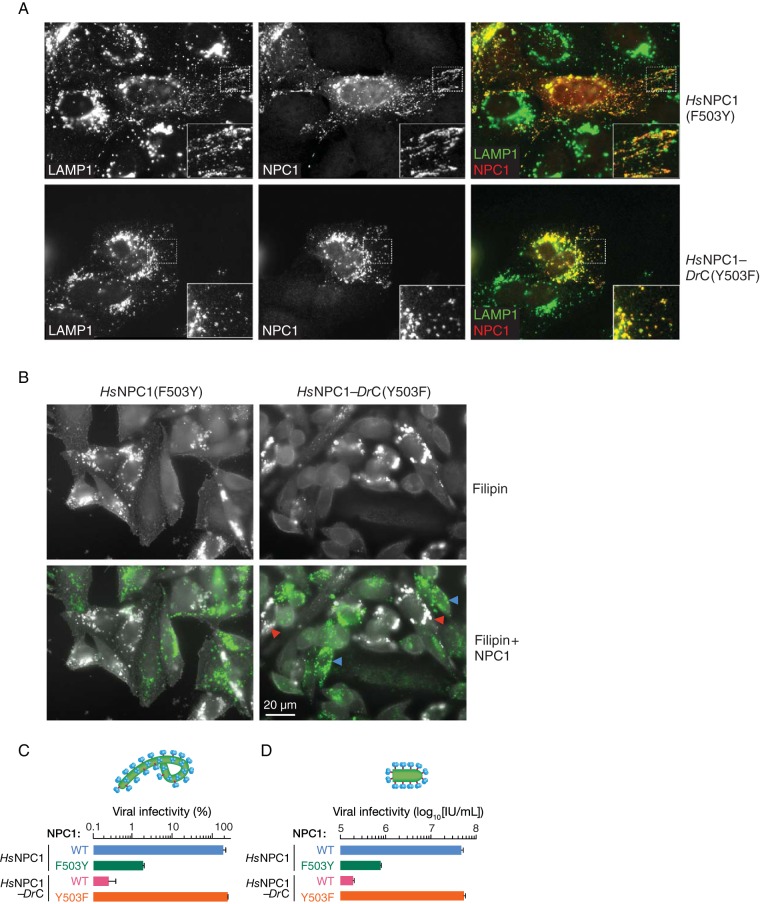
Residue 503 influences the capacity of full-length NPC1 to support EBOV entry and infection. (A) Point mutations at residue 503 were introduced into *Hs*NPC1 and the chimera *Hs*NPC1-*Dr*C (F503Y and Y503F, respectively), and these constructs were transiently expressed in U2OS NPC1^−/−^ cells. NPC1 (red) and a lysosomal marker, LAMP1 (green), were visualized by immunofluorescence microscopy. (B) NPC1-deficient CHO-M12 cells stably expressing either *Hs*NPC1 (F503Y) or *Hs*NPC1-*Dr*C (Y503F) were stained with filipin to visualize unesterified cholesterol. Top panel, filipin staining. Bottom panel, cells immunostained with anti-Flag antibody for NPC1 expression (green). Cholesterol-laden cells are marked with red arrowheads. Blue arrowheads indicate cells that are functional at cholesterol clearance. (C) CHO-M12 cells stably expressing the NPC1 proteins indicated were exposed to authentic virus (multiplicity of infection of 3), scored at 72 h postinfection, and normalized to *Hs*NPC1 WT infectivity. (D) Infection by VSV-EBOV GP, calculated by manual counting of eGFP-positive cells. IU/ml, infectious units per milliliter. Means ± standard deviations (*n* = 4) from a representative experiment are shown in each panel.

To examine the consequences of the 503(F↔Y) sequence change for the cellular and viral receptor functions of NPC1, we introduced the F503Y and Y503F mutations into full-length WT *Hs*NPC1 and the *Hs*NPC1-*Dr*C chimera, respectively, and expressed them transiently in U2OS NPC1^−/−^ cell lines. Immunostaining of NPC1 in these cell lines revealed colocalization with LAMP1 for both WT *Hs*NPC1 and *Hs*NPC1-*Dr*C (Fig. 8A and 3A). Furthermore, filipin staining showed little or no cholesterol accumulation in CHO-M12 cells stably expressing *Hs*NPC1(F503Y) or *Hs*NPC1-*Dr*C(Y503F) (Fig. 8B). Therefore, the F503Y and Y503F mutations do not substantially affect the folding, endosomal delivery, and cholesterol clearance function of NPC1.

Finally, we challenged cell lines expressing the 503(F↔Y) NPC1 mutants with authentic EBOV and rVSV-EBOV GP (Fig. 8C). The capacities of both authentic and surrogate viruses to enter and infect these cells were fully congruent with the results of the GP-binding experiments. The viper→human Y503F mutation afforded the complete restoration of viral infection in cells expressing the *Hs*NPC1-*Dr*C chimera (≈3 log_10_ unit increase). Reciprocally, the human→viper F503Y mutation reduced viral infection in cells expressing *Hs*NPC1 by ≈2 log_10_ units. Thus, the infection data correlate with the GP_CL_-domain C-binding data, demonstrating that switching the residue at position 503 changes the ability of human and Russell’s viper NPC1 domain C to bind EBOV GP_CL_, thereby determining the ability of these NPC1 proteins to be used as EBOV receptors.

### A bulky, hydrophobic amino acid residue at position 503 favors EBOV GP_CL_-NPC1 domain C binding.

**FIG 9:**
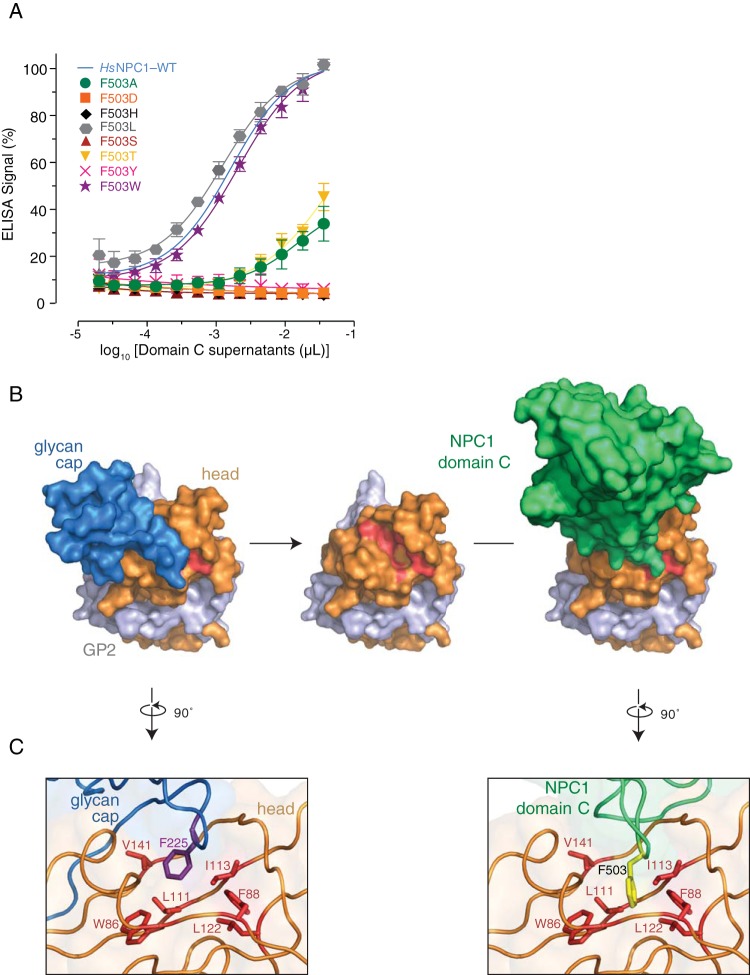
A bulky, hydrophobic residue is required at position 503. (A) The F at NPC1 residue 503 mutated to A, D, H, L, S, T, Y, or W and tested for binding to EBOV GP_CL_ by ELISA. (B) Structure of EBOV GP monomer with GP1 (orange) and GP2 (gray) and the glycan cap (blue) occluding the NPC1-binding site (residues identified as critical for binding are colored red) (PDB identifier 3CSY [28]). Proteolytic removal of the glycan cap and mucin domain (not shown) in host cell endosomes unmasks this site, allowing binding of NPC1 domain C (green) (PDB identifier 5F1B [26]). (C) Comparison of the interaction between residues W86, F88, L111, I113, L122, and V141 (red) in the GP1 hydrophobic trough and F225 (magenta) of the glycan cap (PDB identifier 3CSY [28]), left, versus F503 (yellow) of NPC1 domain C (PDB identifier 5F1B [26]), right.

To determine the mechanism by which the change in NPC1 residue 503 controls binding of *Hs*NPC1 to EBOV GP_CL_, we engineered a series of NPC1 domain C proteins bearing amino acid residues with divergent physicochemical properties at position 503. Examination of these mutants by GP_CL_-binding ELISA revealed that binding avidity was generally correlated with amino acid size and polarity (Fig. 9A). Specifically, residues with bulky, hydrophobic side chains (L and W) afforded GP_CL_-NPC1 binding at WT levels, whereas residues with polar side chains (D, H, and S) abrogated binding. Binding was greatly reduced, but detectable, with A and T at residue 503. The recently solved structure of EBOV GP_CL_ bound to NPC1 domain C shows that residue 503 inserts into the hydrophobic trough of EBOV GP_CL_ (28, 29), similarly to residue F225 of the EBOV glycan cap (Fig. 9B and C) (26).

### The tyrosine residue at position 503 controls EBOV GP_CL_-binding function in reptile NPC1 domain C orthologs.

**FIG 10:**
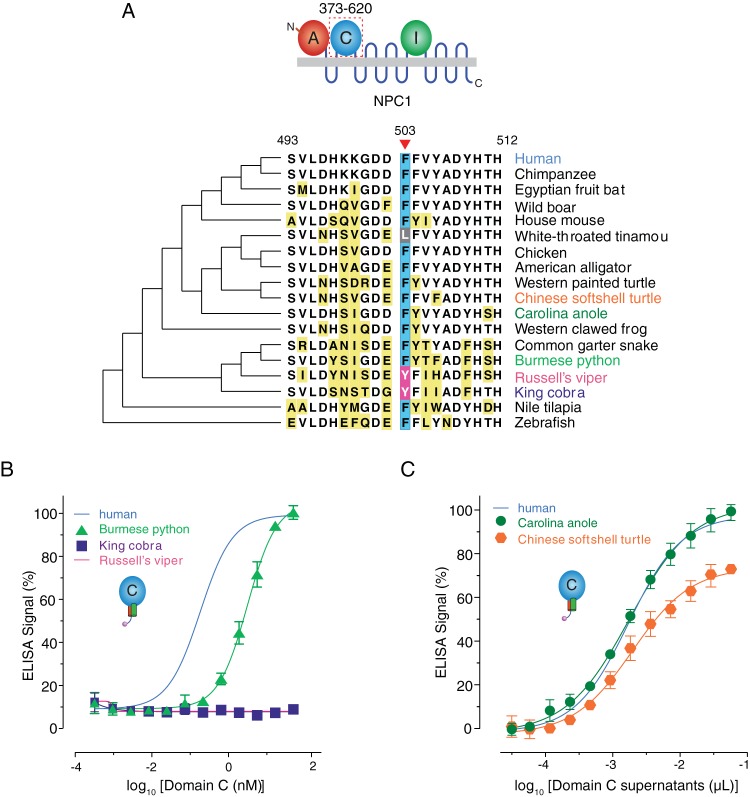
The tyrosine residue at NPC1 position 503 is unique to the Russell’s viper and king cobra *NPC1* orthologs. (A) Alignment of sequences flanking residue 503 (red arrowhead) in domain C from divergent NPC1 orthologs. Residues different from the human sequence are highlighted in yellow. F503 is shaded blue, and Y503 is shaded pink. (B and C) Binding of NPC1 domain C proteins from snakes (B) and other reptiles (C) to EBOV GP_CL_ as determined by ELISA.

Finally, we asked if our findings had implications for host cell range in other vertebrates, especially reptiles, which appear to be refractory to infection by EBOV (8, 30). An alignment of available NPC1 domain C sequences from a panel of vertebrate species revealed that, although there exist a number of differences in amino acid sequence around residue 503, the F at this position is itself very well conserved among vertebrates, with only two *NPC1* orthologs—those of the Russell’s viper and king cobra (*Ophiophagus hannah*)—encoding a Y at this position (Fig. 10A). Interestingly, the predicted NPC1 polypeptide sequences of two additional snakes, the Burmese python (*Python bivittatus*) and the common garter snake (*Thamnophis sirtalis*), show an F at position 503 (Fig. 10A). To investigate the GP_CL_-binding capacities of the snake NPC1 orthologs, we expressed and purified soluble NPC1 domain C proteins for the king cobra and Burmese python and tested them for binding to EBOV GP_CL_. The capacity of these proteins to bind to EBOV GP_CL_ was concordant with the identity of the residue at NPC1 codon 503. Thus, king cobra NPC1 domain C(Y503) resembled viper NPC1 domain C in its inability to bind to EBOV GP_CL_, whereas Burmese python NPC1 domain C(F503) readily bound to EBOV GP_CL_ (Fig. 10B). We tested two more reptilian NPC1 orthologs—from Chinese softshell turtle and Carolina anole (both carrying F503)—and found that they could all bind to EBOV GP_CL_ (Fig. 10C). These findings provide additional evidence that *NPC1*-encoded residue 503 influences the cellular host range of EBOV at the level of virus-receptor recognition and raise the possibility that sequence differences at this position influence the susceptibility of reptiles to filovirus infection in nature.

## DISCUSSION

The essential entry receptor NPC1 is the first known molecular determinant of the cellular host range of EBOV and other filoviruses (25, 26). In this study, we uncover one mechanism by which NPC1 imposes a species-specific barrier to EBOV infection. We show that reptilian cells derived from the Russell’s viper, *Daboia russellii*, are largely resistant to EBOV entry and infection because of the presence of a Y residue at position 503 in NPC1, whereas the NPC1 orthologs of most other types of animals, include humans, carry a highly conserved F residue. Unexpectedly, toggling this residue between F and Y in either human or Russell's viper NPC1 backgrounds switched each protein’s ability to act as an EBOV receptor. NPC1’s crucial housekeeping function—distribution of cholesterol from the endo/lysosomal compartment to other cellular membranes—remained unaffected by these changes. Thus, our work identifies a genetic determinant in NPC1 that controls its viral receptor function, and consequently host susceptibility to EBOV infection, in a manner that is selective and yet transferable between highly divergent NPC1 orthologs.

The determinative F503→Y change is located in NPC1’s second luminal domain (C), which directly binds to a cleaved form of the EBOV entry glycoprotein (GP_CL_) during viral entry (22, 23, 26). Here, we found that F503→Y renders cells nonpermissive to EBOV infection because it reduces the apparent binding affinity of GP_CL_ for NPC1 domain C by more than 1,000-fold. What mechanism might account for this extraordinary effect of a single hydroxyl group on virus-receptor interaction?

The recently solved structure of the EBOV GP_CL_ bound to NPC1 domain C reveals that F503 in human NPC1 domain C inserts deeply into the hydrophobic GP_CL_ trough during GP-NPC1 interaction, in a manner that resembles the interaction of F225 in the GP glycan cap with the GP_CL_ trough in uncleaved GP (Fig. 9B and C) (26). The introduction, at position 503, of a polar hydroxyl group (Y) or other polar side chains (D, H, S, and G) is likely to be energetically unfavorable, thereby reducing the affinity of GP_CL_-NPC1 binding.

We recently demonstrated that residue 502 in NPC1 was under positive selection in bats and was responsible for the reduced susceptibility of African straw-colored fruit bat cells to EBOV infection (25, 31). Since none of the bat species genes encodes a Y at position 503 in NPC1, there was no observed signature of positive selection at this residue. The structure rationalizes the effect of these residues on GP-NPC1 binding, as both are located in the α4-α5 loop of NPC1 domain C that directly interacts with EBOV GP_CL_ (“loop 2” [26]) (Fig. 9C).

It is unclear what relationships, if any, exist (or have existed) in nature between filoviruses and snakes or other reptiles. Experimental infections of wild-caught reptiles and amphibians by Swanepoel and colleagues (30) showed a general refractoriness to EBOV infection or replication, but minimal titers were recovered on a few occasions from the brown house snake (*Lamprophis fuliginosus*). Following outbreaks of the Ebola virus relative Marburg virus (MARV) at the mine in Kitaka Cave, the nearby “Python Cave” in Queen Elizabeth National Park in Uganda (32, 33), and the Goroumbwa Mine in the Democratic Republic of the Congo (31), a number of Egyptian fruit bats were found to be infected with MARV (5, 6, 24, 34). Unfortunately, though the African rock python (*Python sebae*) and forest cobra (*Naja Melanoleuca*) are part of the fauna in these locations, there were no reports on investigations of snakes from these caves for filovirus infection (6, 22, 31). Nevertheless, our finding that two snake NPC1 orthologs are nonpermissive to filovirus entry and infection due to a single amino acid change leads us to speculate that this change was an adaptation to reduce infection by a filovirus, thereby increasing host survivability. More-extensive wildlife sampling coupled with genetic and functional analysis of host-virus interactions associated with filovirus infection may uncover additional evidence for evolutionary arms races between filoviruses and multiple types of animals (bats, reptiles, and rodents).

## MATERIALS AND METHODS

### Cells.

Vero grivet HEK 293T and U2OS cells were maintained in high-glucose Dulbecco’s modified Eagle’s medium (DMEM; Thermo Fisher Scientific, Waltham MA) supplemented with 10% fetal bovine serum (FBS; Atlanta Biologicals, Flowery Branch, GA) and 1% penicillin-streptomycin (Thermo Fisher Scientific) at 37°C and 5% CO_2_. U2OS NPC1^−/−^ cell lines were generated by CRISPR/Cas9 genome editing as previously described (27) and transiently transfected with NPC1 constructs for colocalization experiments. Chinese hamster ovary (CHO) cells were maintained in DMEM–Ham’s F-12 medium (50/50 mix) (Thermo Fisher Scientific) supplemented with 10% FBS at 37°C and 5% CO_2_. Cell lines were generated by a retroviral transduction system, as previously described (22), to stably overexpress the NPC1 constructs in CHO-M12 cells, which contain a deletion in the *NPC1* locus (24). FreeStyle 293-F cells were maintained in Gibco FreeStyle 293 expression medium (Thermo Fisher Scientific) at 37°C and 8% CO_2_.

### NPC1 constructs.

NPC1 domain C sequences (residues 373 to 620) flanked by sequences that form antiparallel coiled coils as previously described (35) were cloned into the pcDNA3.1(+) vector. Constructs made included glycosylation mutants in *Hs*NPC1 domain C (L414N+D416T, K498N+G500S, and N598A), while those in *Dr*NPC1 domain C were N414A, N498A, and R600T. *Dr*NPC1 domain C chimeras were made by substituting these residues for human residues 373 to 475 (chimera 1), 476 to 536 (chimera 2), 537 to 620 (chimera 3), 493 to 502 (chimera 4), 502 to 512 (chimera 5), and 513 to 522 (chimera 6), and the point mutations made were E502D, Y503F, I505V, H506Y, F509Y, and S511T. The constructs were then transiently transfected into HEK 293T cells, and the supernatant with secreted protein was harvested after 72 h and used in ELISAs. Purified proteins were made by transfecting FreeStyle 293-F cells in suspension, harvesting cells 72 h posttransfection, and purifying them by incubation with His-60 nickel resin. The proteins were eluted at 500 mM imidazole and pH 7.6 and dialyzed into 50 mM 2-(*N*-morpholino)ethanesulfonic acid (MES), 150 mM NaCl, pH 5.5. Domain C chimeras in the full-length NPC1 were generated by seamlessly replacing the domain C sequences in *Hs*NPC1. The constructs were subcloned into the pBABE-puro retroviral vector and stably transfected into CHO-M12 cells by retroviral transduction, as previously described (22). All constructs possessed N-terminal Flag tags.

### VSV pseudotype infections.

Replication-incompetent vesicular stomatitis virus Indiana (VSV) pseudotypes encoding enhanced green fluorescent protein (eGFP) in the first position and EBOV GP in place of VSV G were made as previously described (9, 36). EBOV GPΔMuc matches the EBOV/H.sapiens-tc/COD/1976/Yambuku-Mayinga isolate amino acid sequence (GenBank accession number AF086833) but lacks the mucin-like domain (Δ309–489; ΔMuc) (37). Unless otherwise indicated, virus titers were determined on Vero grivet monkey cells by manual counting of eGFP-positive cells. Cleaved EBOV GP (GP_CL_) was generated *in vitro* using the bacterial protease thermolysin (250 µg/ml) (Sigma-Aldrich, St. Louis, MO) for 1 h at 37°C as described previously (38, 39), and the reaction was stopped by adding the metalloprotease inhibitor phosphoramidon (1 mM) (Sigma-Aldrich).

### Authentic Ebola virus infections.

CHO cells, seeded in black Cellcoat 96-well plates (Greiner Bio-One, North America, Monroe, NC) were incubated with Ebola virus/H.sapiens-tc/COD/1995/Kikwit-9510621 at the indicated multiplicity of infection in a biosafety level 4 (BSL-4) laboratory located at USAMRIID. Following a 1-h absorption, virus inoculum was removed and cells were washed once with phosphate-buffered saline (PBS). Cells were then incubated at 37°C, 5% CO_2_, and 80% humidity for 72 h, at which time the cells were washed once with PBS and submerged in 10% formalin prior to removal from the BSL-4 laboratory. Formalin was removed, and cells were washed 3 times with PBS. Cells were blocked by adding 3% bovine serum albumin (BSA)-PBS to each well and incubating the cells at 37°C for 2 h. Cells were incubated with EBOV GP-specific monoclonal antibody (MAb) KZ52, diluted to 1 µg/ml in 3% BSA-PBS, at room temperature for 2 h. Cells were washed 3 times with PBS prior to addition of goat anti-human IgG-Alexa Fluor 488 (Thermo Fisher Scientific) secondary antibody. Following a 1-h incubation with secondary antibody, cells were washed 3 times prior to addition of Hoechst 33342 (Thermo Fisher Scientific) diluted in PBS. Cells were imaged and percentages of virus-infected cells were calculated using the Operetta high-content imaging system (PerkinElmer, Waltham, MA) and Harmony high-content imaging and analysis software (PerkinElmer).

### GP_CL_-NPC1 domain C capture ELISA.

Normalization of NPC1 domain C supernatants and proteins was carried out as previously described (25): resolution on SDS-PAGE gels followed by immunoblotting with anti-Flag primary antibody (Sigma-Aldrich) and anti-mouse Alexa-680 secondary antibody (Thermo Fisher Scientific) and quantification on the Li-Cor Odyssey imager (Li-Cor Biosciences, Lincoln, NE). Capture ELISAs were also performed as previously described (22, 25). Briefly, high-binding 96-well ELISA plates (Corning, Corning, NY) were coated with KZ52 (40) (2 µg/ml in PBS) and then blocked using PBS containing 3% bovine serum albumin (PBSA). Pseudotyped EBOV was cleaved with thermolysin (250 µg/ml) at 37°C for 1 h and captured on the plate. Unbound virus was washed off, and serial dilutions of either Flag-tagged purified soluble NPC1 domain C (domain C; 0 to 40 µg/ml) or supernatants from transient transfections of the NPC1 constructs on HEK 293T cells were added. Bound domain C was detected by a horseradish peroxidase-conjugated anti-Flag antibody and Ultra-TMB substrate (Thermo Fisher). EC_50_ values were calculated from binding curves generated by nonlinear regression analysis using Prism (GraphPad Software, La Jolla, CA). Binding ELISAs were done in duplicate and in at least two independent experiments. All incubation steps were done at 37°C for 1 h or at 4°C overnight.

### Immunofluorescence.

Imaging was performed in U2OS or CHO cells grown on 12-mm coverslips and fixed with 4% paraformaldehyde. For antibody staining, the coverslips were incubated with an anti-Flag antibody (Sigma-Aldrich) in PBS containing 0.1% Triton X-100 and 1% BSA. Detection was by incubation with Alexa 488-conjugated secondary antibodies (Thermo Fisher Scientific). For filipin staining, the coverslips were stained with 50 μg/ml of *Streptomyces filipinensis* filipin III complex (Sigma-Aldrich) in PBS for 1 h. Coverslips were mounted on glass slides using ProLong antifade reagent (Thermo Fisher Scientific), and images were acquired with an inverted fluorescence microscope equipped with a 63× high-numerical-aperture oil objective.
